# Trace: A research media player measuring real-time audience engagement

**DOI:** 10.3758/s13428-024-02522-0

**Published:** 2025-01-06

**Authors:** Ana Levordashka, Mike Richardson, Rebecca J. Hirst, Iain D. Gilchrist, Danaë Stanton Fraser

**Affiliations:** 1 Department of Psychology, University of Bath, Claverton Down, Bath, BA2 7AY UK; 2 Open Science Tools, Nottingham, UK; 3 School of Psychological Science, University of Bristol, Bristol, UK

**Keywords:** Face tracking, Attention, Engagement, Audiences, Research application

## Abstract

Measuring attention and engagement is essential for understanding a wide range of psychological phenomena. Advances in technology have made it possible to measure real-time attention to naturalistic stimuli, providing ecologically valid insight into temporal dynamics. We developed a research protocol called *Trace*, which records anonymous facial landmarks, expressions, and patterns of movement associated with engagement in screen-based media. Trace runs in a standard internet browser and resembles a contemporary media player. It is embedded in the open-source package PsychoJS (the JavaScript sister library of PsychoPy) hosted via Pavlovia, and can be integrated with a wide range of behavioral research methods. Developed over multiple iterations and tested with over 200 participants in three studies, including the official broadcast of a major theatre production, Trace is a powerful, user-friendly protocol allowing behavioral researchers to capture audience attention and engagement in screen-based media as part of authentic, ecologically valid audience experiences.

**Supplementary information:**

The online version contains supplementary material available at 10.3758/s13428-024-02522-0.

## Introduction

The study of attentional engagement has been a central topic in the fields of cognition and behavior. The human ability to pay attention and selectively engage plays a crucial role in fundamental psychological processes such as perception (Luck & Ford, 1998; Spence & Driver, 1998), decision-making (Orquin & Mueller Loose, 2013), social cognition (Mundy & Newell, 2007), and memory (Chun & Turk-Browne, 2007; Desimone, 1996). Over the past century, methods to measure attention have progressed quickly (for a review, see Driver, 2001), from seminal work on the renowned ‘cocktail party effect’ (Broadbent, 1954) right up to modern computational modeling to investigate underpinning neural correlates (Peñaloza & Ogmen, 2022). However, despite several relevant psychophysical, psychometric, and observational paradigms, measuring attention remains non-trivial. On the one hand, common methods such as reaction time-based tasks (e.g., the Posner Cueing Paradigm; Posner et al., 1984; Posner, 1980) often lack ecological validity, and on the other, prevalent questionnaire-based measures may have a poor temporal resolution (i.e., they are issued post-test or post-stimulus presentation, not in real-time) or high variation based on the mode of administration (Bowling, 2005).

In recent years, advances in technology have made it possible to measure attention in real time while individuals are exposed to stimuli such as videos, images, and sounds (Lopez-Palma et al., 2018; Treder et al., 2014). Applying these approaches to entertaining content, such as movies or television programs, allows researchers to measure engagement of attention in real-time with an activity that most individuals already participate in for many hours per week: watching screen-based media (Vioque et al., 2000). Many of these methods still require specialized equipment, for example, electroencephalography (EEG) headsets, eye-trackers, or specialized cameras, meaning experimentation is often limited by logistics so are restricted to a laboratory environment, or even the presence of such equipment may influence engagement (Tschacher et al., 2021).

Engaging with films, television, and other screen-based media also offers an ideal test bed to explore other crucial cognitive and behavioral processes beyond attention. These mediums are often the source of highly emotional experiences (Carvalho et al., 2012; Gross & Levenson, 1995). The death of a beloved character causes the sensation of real loss (Degroot & Leith, 2018) or watching a favorite sports team can elicit powerful joy and identity (Branscombe & Wann, 1991). In research settings, emotionally valanced clips are often used as stimuli to provoke an emotional response (Dennis & Solomon, 2010; Ellard et al., 2012; Fernández-Aguilar et al., 2019). However, capturing these processes ‘in the wild’ is extremely difficult, with researchers typically defaulting to post-test self-report methods. Recent work has begun to explore the use of machine learning and computer vision to detect expressions to provide a real-time measure of emotion (Chen et al., 2017; Michel & El Kaliouby, 2003).

Defining audience engagement is complex, and as such, this has led to multiple different definitions within and between research fields. In this article, we use the term engagement to describe the level of attention or mental involvement given to a piece of screen-based media. In recent studies, we categorized four main levels of engagement: an audience member could be distracted or disengaged, attentive or engaged (but not deeply focused), reflecting on the content (e.g., task-related mind-wandering), or immersed or deeply engaged (Levordashka et al., 2023). Past work has suggested that immersion is a very high level of engagement, or a ‘state of deep mental involvement, accompanied by a reduced awareness of the physical world’ (Agrawal et al., 2020, p. 413). Neuroimaging investigations also confirm the activation of attentional networks (along with memory regions, reward systems, and mentalizing) during moments of engagement (Ki et al., 2016; Ohad & Yeshurun, 2023; Song et al., 2021). The recent two-competitor model of attention in immersion has posited that immersion is represented as competing dimensions of attention between either the induced (through sensory or narrative means) or the actual physical reality and can be assessed through neurophysiological and psychophysical investigations of attention (Strauss et al., 2024).

The physical movements of an audience can provide a unique insight into the engagement of those audience members. For example, in dance, past work has demonstrated that collective stillness of an audience may indicate engagement (Theodorou et al., 2016, 2019). Conversely, other experiments into audience movement might suggest that increased movement may indicate engagement, such as the entrainment of breathing to a rhythm of a performance (Bachrach et al., 2015) or involuntary face movement in response to a surprising or fearful event in film or television (Hubert & de Jong-Meyer, 1990). While context is important when interpreting audience movement, a promising avenue lies in collective or synchronous movement, inspired by studies into physiological synchrony as a robust indicator of engagement (Hammond et al., 2023; Madsen & Parra, 2022; Pérez et al., 2021; Stuldreher et al., 2023). Furthermore, facial expression synchrony predicts engagement in theatre audiences (Oakes et al., 2024). Investigations into audience movements warrant further investigation, as there are still many unanswered questions surrounding behavioral movement in response to the process of cognitively engaging with content. However, the acquisition of such movement data can be difficult to obtain, often requiring expensive camera setups and data processing pipelines (Theodorou et al., 2019). While audience movements have been explored in response to dance performance (Bachrach et al., 2015; Theodorou et al., 2016, 2019; Upham et al., 2024), they have been examined to a much lesser extent in film and television. Considering audience synchrony more holistically, evidence suggests a trend towards an engaged audience being more synchronized than a less engaged audience. For example, Hammond et al. (2023) found that heart rate synchrony was positively correlated with self-reported engagement. While measures such as heart rate synchrony appear to be a valid real-time measure of engagement at a collective audience level, there are issues of scalability and they cannot easily be applied to remote studies.

There are certainly benefits to using real-time measures of audience engagement. At present, the vast majority of audience methods employed by researchers are questionnaire-based (for a review, see Richardson et al., 2024). Questionnaires do have their advantages. They can be easily conducted remotely, at scale, and for little expense; these reasons are perhaps why they dominate the landscape of audience research. However, questionnaires rely on memory processes and may be influenced by the primary and recency effect (Hands & Avons, 2001; Li, 2009) and specifically, the ‘peak-end’ effect (Latulipe et al., 2011). Additionally, long form content such as TV series or some films, may not easily be completely captured by a set of numerical scales. In sum, measuring an audience’s experience is non-trivial and while different methods may provide some insights to parts of the experience, it is only through the triangulation of multiple methods that we can begin to build a better picture. However, many of these methods are invasive or intrusive to either the audience or to the performance and could reduce immersion and engagement simply by collecting that information.

This paper presents a novel approach to measuring real-time engagement in screen-based media by developing a research protocol called Trace. Trace is a web-based media player that uses face tracking to measure engagement in media content. We argue that Trace has several advantages over traditional methods of measuring engagement with screen-based content. Firstly, Trace has high *ecological validity*. It was successfully used by a diverse sample of participants, including in the public broadcast of a major theatre production. Through its integration with the JavaScript sister library of PsychoPy, PsychoJS, and Pavlovia (Bridges et al., 2020; Peirce et al., 2019) and use of face-api (*face-api.Js*, n.d.) Trace can be used alongside a wide range of experimental procedures to ensure *internal validity* and can be easily added to existing laboratory-based or field studies requiring only a laptop and an internet connection. Moreover, Trace operates anonymously within a web browser, making it easy to use and *accessible* to a wide range of participants, including watching content in a naturalistic setting (e.g., at home) if required.

Trace has the functionality to allow researchers to detect mind wandering and inattention during content consumption. For example, the links between fidgeting and periods of inattention have been demonstrated via self-report questionnaire (Carriere et al., 2013), but such methods may be limited temporally and via probes that ask report to report whether they are paying attention (Seli et al., 2016), however, probes typically interrupt the trial and/or stimulus presentation potentially breaking feelings of immersion, engagement, and transportation.

We discuss the technical details of Trace and describe how it has been used in existing projects to demonstrate its feasibility and potential. Trace unlocks the ability for behavioral science researchers to gain a nuanced understanding of how attention and other cognitive functions operate in real-world contexts.

### Design and implementation

Trace uses web camera and face detection to anonymously trace the position of the user’s face, capturing (a) 68 key facial landmarks (e.g., inner eye corners, the tip of the nose) and (b) seven facial expressions (*happy, sad, angry, disgusted, surprised, fearful, neutral*), inferred through a multi-task cascaded convolutional neural network. Although Trace has the functionality to detect basic emotions, this functionality has not been tested and the focus of the present article is limited to movement detection. The facial coordinates can be used to compute an overall movement metric associated with low engagement, as described in (Levordashka et al., 2023). The movement and facial expressions data are time synchronized with media content to allow for individual differences in streaming due to lag or pausing of the content.

#### Face detection

Face landmark detection is accomplished through face-api (*face-api.Js*, n.d.), a JavaScript library built on top of TensorFlow, which is a popular open-source machine learning library. Face-api requires no external dependencies and utilizes a WebGL backend. Face-api was trained on a custom dataset, including Facial Expression Recognition 2013 and 300 Faces in-the-Wild (Dumitru et al., 2013; Sagonas et al., 2013). Trace is implemented in the FaceDetector class of PsychoJS (https://github.com/psychopy/psychojs), the JavaScript sister library of PsychoPy (Peirce et al., 2019) and hosted on Pavlovia (Bridges et al., 2020), an online hosting service for studies created in PsychoPy, a widely used application for creating experiments in behavioral science.

#### Participant experience

**Fig. 1:**
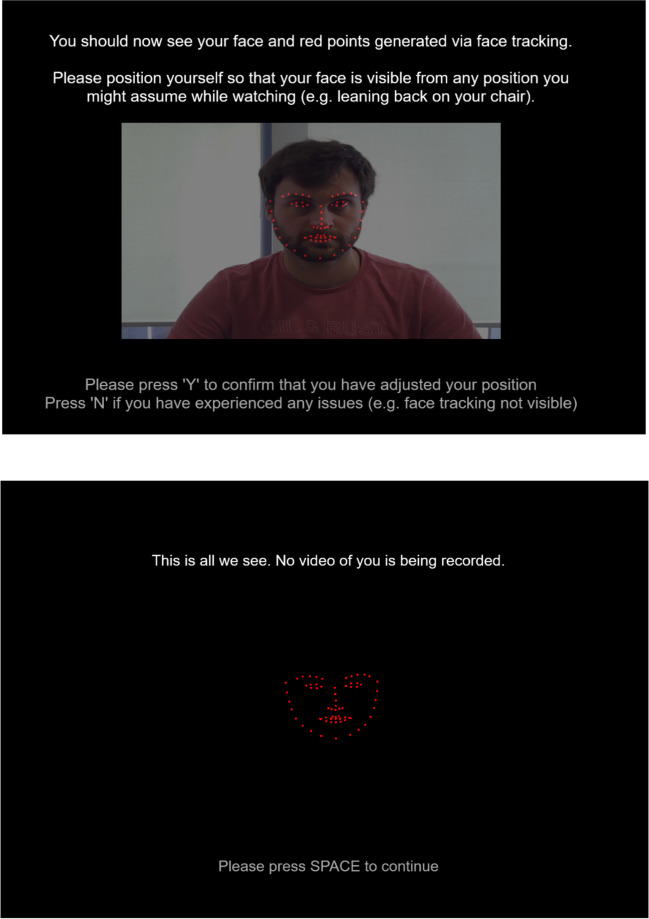
Two screenshots taken during the opening instructions of Trace. *Note.* The top image shows the initial opening of Trace. The participant can see how the landmark points map onto their own face. The bottom image shows the following screen, demonstrating that the raw camera feed is removed from recording and only the landmarks remain

Participants or audiences receive a link. When the link is opened, Trace provides a short introduction, including a request for consent to activate the device’s camera. Trace then starts recording the anonymous facial landmarks and stores them on Pavlovia’s secure server. By default, no video footage is stored or transmitted during the entire process, ensuring anonymity servers (see Fig. 1 for demonstration).

#### Research capabilities

Trace is based on PsychoPy/PsychoJS and can draw on the package’s versatile infrastructure for blocking, randomization, stimulus presentation, and behavioral measures, such as reaction times. Media content can be embedded directly as a media file or hosted on YouTube and streamed to the experiment. It can also be hosted externally, with Trace redirecting participants to an external page and running in the background (see use case 2).

#### Data

Within Trace, face-api data are logged for each frame and appear alongside other data recorded in the experiment in csv format (the default data saving format for PsychoJS). Data are then stored on the Pavlovia hosting server. The frame rate at which data are time-stamped will be device-dependent (i.e., dependent on the frame rate). The output from face-api consists of the following variables:

- *landmarks* are outputted as a nested list with 68 comma-separated parameters, each containing comma-separated* x*–*y* coordinates (in pixels).
- *emotionDetection* are seven continuous numeric variables (*happy, sad, angry, disgusted, surprised,* *fearful, neutral)*, each representing the probability (range 0 to 1) that the respective emotion is detected.
- *unixtime* is the precise time at which data were sampled, recorded as a UNIX timestamp (date and time representation, which measures time by the number of seconds that have elapsed since January 1, 1970, at UTC, with millisecond precision). UNIX timestamp is widely used in computing and can make Trace compatible with various software and hardware (e.g., commercial heart rate monitors such as the Empatica E4 (Empatica, USA).
- *yt_time* (when using YouTube content) is the current time in the video, requested from YouTube’s API via *getCurrentTime*(). This variable can be used to align face tracking and other PsychoJS data with the content being received by a participant, preventing inconsistency due to time lag and buffering.

#### Setting up

To set up Trace in Pavlovia, the researcher or event organizer must create an account, clone, and adapt the base experiment. Instructions are provided in the repository and are available in Appendix 1.

### Evaluation

Trace was developed over multiple iterations to optimize its performance and usability. We report user studies with over 200 international participants who consumed screen-based content of up to 3 h in duration (see Table 1). Trace was used in conjunction with electronic and paper-based follow-up questionnaires, experience sampling, and physiological measurements. The studies were part of different projects; their main results are not reported here. However, we do provide a brief overview of the methodological approach for context and references to the primary sources. We report information on data quality and linking. All research was performed in accordance with the Declaration of Helsinki and reviewed and approved by the University of Bath Department of Psychology Research Ethics Committee (20–202).

**Table 1:** Overview of trace’s use cases

Use Case 1 Remote academic research	Use Case 2 In-person audience research	Use Case 3 Remote audience research
131 participants (at home) Research Panel (Prolific), paid 25-min video, hosted internally Measures Face-tracking Experience sampling NES questionnaire	42 participants (lab) Ticket holders, paid 3-h livestream, hosted externally Measures Face-tracking Empatica HR monitor NES questionnaire	6 participants (at home) Ticket holders, volunteer 3-h livestream, hosted externally Measures Face-tracking NES questionnaire

NES = Narrative Engagement Scale (Busselle & Bilandzic, 2009); HR = heart rate.

### Use Case 1: Academic research on audience engagement

Trace was necessitated by and developed for a research project on non-verbal signatures of audience engagement. Conceptualized prior to the COVID-19 pandemic, the research aimed at capturing audience experience and behavior at live events, such as theatre plays and concerts, produced by partnering Arts & Culture institutions. Worldwide lockdowns in response to the pandemic prevented live events and in-person data collection. We therefore began to explore alternative options for conducting audience research remotely. During the development of the tool, it became apparent that this method would have beneficial functionality outside the pandemic, too, allowing for remote research on audience movement. This use case and main results are detailed in Levordashka et al. (2023). Data concerning the study is available here: https://osf.io/vxbj7/?view_only=8fbfca96a01e4d79ad50bbe448c03c65. The stimulus content was a 30-min segment of The Bullet and the Bass Trombone by sleepdogs (*Sleepdogs _ The Bullet And The Bass Trombone*, n.d.).

#### Participants

Participants were recruited from an academic research panel (Prolific) and reimbursed at a rate of £8 per hour. The final sample consisted of 215 participants, with an average age of 30 years, with standard deviation 11 years. Fifty percent of participants were female, 47% male, and three nonbinary. The sample was somewhat ethnically diverse (66% White, 21% Black, 5% Mixed/Multiple, 3% Hispanic, and 3% Asian).

#### Method

Trace was used to stream a 30-min segment of a theatre play to remote participants’ home devices. Operating systems included Windows (87%) and Mac OS (12%); browsers were Chrome (78%), Firefox (14%), Edge (5%), and Opera (3%); screen resolution ranged from 1080 × 1920 to 2560 × 1440. Motion tracking was used in conjunction with an experience sampling measure and a follow-up experience questionnaire hosted on Qualtrics. Video recordings from the participants’ web cameras were stored alongside face-tracking data. In the study, participants were trained to press one of four keys to indicate, when prompted, if they were feeling immersed, engaged, reflecting, or distracted (and were provided with definitions of these states during the training phase). At the end of the study, participants were asked if they would like to continue watching the piece as well as completing the narrative engagement scale (Busselle & Bilandzic, 2009).

#### Results

The face tracking and experience sampling data were synchronized via PsychoJs’s highly precise (Peirce et al., 2019) internal clock. To connect the external questionnaire, the participant ID assigned in Prolific was passed on as a URL parameter. The total duration of the stimulus content was 1680 s. When down-sampled to 1 s, face-tracking data were available for 78% of seconds (SD = 30), indicating that the participants were present for the majority of duration and date were recorded. Face-tracking data during the final 5 s was recorded for most participants (80%), indicating low attrition. Attrition did not depend on device characteristics or demographics. The average sampling frequency per participant was 5 Hz (i.e., 5 data points per second; SD = 3).

The main findings are detailed in Levordashka et al. (2023). However, we provide a brief overview here. Participants who reported feeling distracted exhibited more head movement compared to immersed or engaged. Additionally, participants who reported that they would not like to continue watching, given the choice, also exhibited more head movement according to the facial landmark tracking. Head movement was negatively correlated with self-reported narrative engagement.

#### Discussion

Movement data were collected successfully from individuals with a broad range of device specifications and viewing conditions. Since the sample consisted of paid participants on a research panel, the positive reception and completion rate may have been motivated by the monetary reward and prior experience with academic research studies. The following use case demonstrates the reliability of Trace with longer, externally hosted content and a sample from the public. The participants took part in their own homes and on their own devices, demonstrating that Trace can be used in a natural remote setting.

## Use Case 2: In-person audience research

Trace was deployed in an audience research collaboration between universities and Bristol Old Vic theatre in Southwest England during a run of the play ‘Drive Your Plow Over the Bones of the Dead’ produced by Complicité, directed by Simon McBurney, based on the novel of the same name by Olga Tokarczuk (2019). Movement and physiological responses were recorded from real audience members attending a limited-run Live Broadcast of a major theatre production. The data are available here: https://osf.io/mvazp/?view_only=8fbfca96a01e4d79ad50bbe448c03c65. The experience data were compared to a sample of ca. 100 individuals who watched the performance live (Carter et al., 2024).

### Participants

The study took place during each Live Broadcast on three separate nights. All ticketholders (ca. 80 per night) were invited via e-mail, and 14 participants per night were selected on a first-come, first-serve basis. It is worth noting that we only recruited from people who had already bought a ticket for the livestream and offered to pay their expenses and offered them a reimbursement of a £30 theatre voucher, if they would like to come into the theatre in person on the night of the live-stream. This ensured that our sample was a valid representation of individuals who would typically watch a live-stream of a Bristol Old Vic production.

### Method

An experimental lab was set up for the purpose of the study, with individual viewing booths consisting of a laptop and overhead headphones. Participants were fitted with a heart rate monitor (Empatica E4 wristband) and watched the performance on a laptop (Dell Latitude 5320, Intel i5 Processor, running on Google Chrome Web Browser) where Trace was actively recording. This lab was constructed in the theatre. Therefore, participants still had to come to the theatre in person, mingle with theatergoers who were watching the performance in the auditorium, and otherwise share all contextual experiences of going to watch a performance at the theatre. The only difference between our participants and ‘regular’ theatergoers, was that when the audience took their seats, our participants entered our temporary lab and watched the performance on a laptop running Trace.

### Results

Three-hour-long recordings were obtained. Physiological data and face-tracking data were linked through UNIX timestamps, which were recorded by both the Empatica E4 device and Trace. The Live Broadcast was hosted on a proprietary platform, which could not be incorporated directly into Trace. Therefore, Trace ran in a separate browser window in the background while the participants watched via the streaming platform. To synchronize the Trace measurement data to the media content, we used the audio recording of the live performance and automated transcription to give UNIX timestamps based on the script, which was then matched to UNIX timestamps provided by Trace. Details of the study can be found in (Richardson et al., 2024). In sum, we found that head movement via Trace correlated with movement measured by the wrist, and confirming Use Case 1, we also found that head movement negatively correlated with the Narrative Engagement Scale (Busselle & Bilandzic, 2009).

### Discussion

This research successfully used Trace for substantially longer recordings (3 h). Movement data were successfully linked to heart rate monitor data, via UNIX API. Linking with content required additional operations due to the creative partners’ use of proprietary services. For content hosted on YouTube, the elapsed time is obtained via the API and stored alongside Trace timestamps. While this use case demonstrates that Trace was intuitive to members of the general public, the initial setup was completed in the presence of a facilitator/researcher and thus did not fully demonstrate its remote usability.

## Use Case 3: remote audience research

As part of the research described in Use Case 2, ticketholders watching the live broadcasts at home were invited to participate. Participants were given a link to Trace and instructed to open it in parallel to the streaming service used by the theater.

### Participants

Participation was voluntary, and no incentive was provided. Recruitment was facilitated by a partner institution, and only a small number of people (*n* = 8) were reached. We consider the individuals who opened a link to Trace as participants. Data are available here: https://osf.io/mkrqd/.

### Method

Participants were in their own homes. Instructions were provided in the recruitment materials and an initiation survey. Instructions were to watch the performance using a device with an active web camera, and launch Trace, which opened the website on which the performance was hosted in a new tab and remained running in the background.

### Results

Eight individuals opened the study link. They all followed the instructions and successfully launched Trace. Two participants dropped out during the performance, possibly due to a decision to stop watching. Due to the small sample size, we do not make any attempt at deeper analysis here or anywhere else. We only comment on the deployment of Trace. Raw anonymized data is available here: https://osf.io/mkrqd/.

### Discussion

This use case demonstrates a successful follow-through with the public with a stable 3-h recording at home. However, due to the creative partners’ reliance on proprietary software, content synchronization was reliant on aligning UNIX timestamps from internal clocks. All clocks were synchronized from the same source at the beginning of the performance, but alignment issues may have occurred. This method of alignment is feasible (depending on the reliability of the internal clock of each device). However, we recommend keeping inside one program (e.g., using Trace’s YouTube integration) to ensure reliability. While we cannot fully rule out the possibility that dropouts were technical errors, the study demonstrated a good follow-through rate and stable recordings with home audiences but lacked a larger scale at home deployment. Future work might aim to test Trace’s usability for a larger at home audience.

## Discussion

Trace provides a valuable tool for measuring engagement in real time and capturing ecologically valid audience behavior. This has important implications for the field of behavioral research, particularly for studying dynamic attention and content engagement. By allowing researchers to capture audience engagement during prolonged stimuli, Trace provides a more comprehensive understanding of underlying attentional processes behind engagement and immersion and can facilitate the development of new theories and models.

For remote studies, Trace can also be implemented to provide insight into participant compliance, providing an easy way to detect whether a participant remains present and looking at the screen during an experiment without the need for interrupting attention check questions. While this does not necessarily indicate whether the participant is actually paying attention during a study, it is a useful metric to detect whether a participant is at least present and looking at the screen. Tracking a participant’s movement can also be used to provide a ‘fidget’ metric. Fidgeting can be used as an indicator of dwindling attention but typically relies on either self-report measures for remote studies or special equipment such as a hacked Wii-Balance Board for laboratory studies (Carriere et al., 2013; Farley et al., 2013; Seli et al., 2014). The benefit of Trace for measuring fidgeting is that it can be incorporated into virtually any design in which a participant interacts with a computer interface (for example, see Levordashka et al., 2023).

Furthermore, Trace can be useful for content creators and practitioners, who can use it to understand which aspects of their content capture the user's attention and which ones need improvement. The low technological requirements (i.e., a laptop with a built-in webcam) mean it can be adopted by a wide range of practitioners, adding a valuable component to existing audience experience toolboxes, or offering low threshold access for those with limited resources for measuring their audience. Although Trace does not currently run on mobile or tablet devices, this could be easily implemented in future versions. This could lead to more effective content creation strategies across various creative and industrial levels, resulting in higher levels of engagement and improved user experiences.

The strengths of Trace include its validation in three different contexts with a broad range of participants, providing authentic audience experiences, and being open source. Additionally, the development of Trace supported the open-source project PsychoPy/PsychoJS, which has implemented features based on the work commissioned for Trace.

There are some notable limitations to the tool. Trace requires a web camera, which may not be available on all devices, and the quality of the camera and lighting conditions can affect its performance. The emotional expression estimations are provided by face-api, and their accuracy across a variety of populations has not yet, to our knowledge, been experimentally validated. Testing the accuracy of the reported emotional expressions will be an important direction for future research. Currently, Trace is designed to capture the face of one person, although it is based on the face-api algorithm, which supports the detection of multiple faces, making a multi-user extension viable. Another limitation is that Trace is currently only available on PsychoJS, although since it is based on the open-source package face-api it should also be possible to implement it in other platforms, such as lab.js or jsPsych, as the code is open.

As Trace utilizes a webcam feed, there is a great degree of flexibility in terms of input. Study 2 was conducted in a theatre, where the low ambient lighting was challenging for the inbuilt laptop webcam. However, for future research in similar venues, Trace’s flexible camera input ensures that infrared or low-light webcams are an option. Should multi-user detection be implemented, capturing audiences in cinemas or theatres in groups with a single high-quality wide-angle camera would be quite feasible.

It is worth considering the ethics of using camera-based audience detection for research. Trace has been developed in an academic context and the use-cases demonstrated here all had ethical approval from a relevant UK institution and all participants provided informed consent. However, as Trace is entirely open-source, there exists the possibility that someone could use this tool without acquiring ethical oversight. However, Trace does not store video or audio information to ensure the anonymity of participants. In addition, Trace is a local system using the pre-trained model of face-api to operate; it does not record, capture, or send any information for model training. This is where it offers distinct advantages to other tools such as GPT-4o or Llama. The data captured by Trace is only accessible to the researcher hosting the study. We have designed Trace to be a tool for measuring real-time engagement for researchers conducting studies on screen-based audiovisual media and hope that it will help provide insights into the nature of engagement with a lower resource cost.

Future directions for Trace could include providing real-time feedback on the level of engagement of individuals or groups through a dashboard accessible to the presenter or event organizer. Trace has further yet unvalidated features that warrant further investigation. For example, face-api can be used to provide emotional estimations of the audience, blink rate and gaze can be calculated from the landmark data, and it is possible to detect multiple faces at once for group viewing. Additionally, Trace could explore advanced operationalization of movement, such as pose estimation and movement in three dimensions. Addressing these limitations and continuing to develop new features could make Trace a more powerful tool for measuring engagement in real time and capturing authentic audience experiences. There are many aspects of audience movement behavior that are yet to be fully understood and we hope that Trace can be another tool to help investigate people’s experience of media, particularly for remote research. In our case studies we used Trace as one method among others, including physiological data recordings, self-report validated questionnaires, and qualitative feedback. Further work would help continue investigating how movement metrics triangulate with other measures to provide more holistic insights into the audience experience.

Overall, the development of Trace represents a significant advance in the measurement of attention and has the potential to provide valuable insights into a wide range of psychological phenomena. By offering a user-friendly and versatile application, it can be integrated with a range of behavioral research methods and has the potential to revolutionize the way attention is measured in both research and practice.

## Conclusion

Trace is a promising tool for measuring engagement in screen-based media in real time. The tool accurately captures the user's engagement levels and provides valuable data that can be used to improve media content. Further development of Trace could lead to a more comprehensive understanding of engagement in screen-based media.

## Supplementary Information

Below is the link to the electronic supplementary material.

Supplementary file1 (DOCX 612 KB)

## Data Availability

This paper does not constitute a primary report of any studies or associated data.

## Code availability

All code is available on https://gitlab.pavlovia.org/PsychologyBath/trace

## Declarations

### Open Practice statement

Trace is open source and available freely, along with all code from https://gitlab.pavlovia.org/PsychologyBath/trace. Trace was not pre-registered.
