# Supplementary material for: Trace: A research media player measuring real-time audience engagement
Source: Behav Res Methods. 2025 Jan 6;57(1):44. doi: 10.3758/s13428-024-02522-0 (PMC11703984; doi:10.3758/s13428-024-02522-0)
Supplement: Supplementary file 1 — Supplementary file1 (DOCX 612 KB) [file 13428_2024_2522_MOESM1_ESM.docx]

Appendix 1

**Trace User Guide**

This documentation describes how to deploy and customize Trace using the PsychoPy GUI. We assume familiarity with PsychoPy/Pavlovia.

**Getting started**

To start, [download and install PsychoPy](https://www.psychopy.org/download.html), launch the PsychoPy Builder and [sign into Pavlovia](https://pavlovia.org/docs/designers/signin). Navigate to the tab ‘Pavlovia.org’ > Search Pavlovia, search for `Trace`, and Fork the project . Alternatively, you can fork the project on [GitLabs](https://pavlovia.org/docs/experiments/create-fork): https://gitlab.pavlovia.org/PsychologyBath/trace.

Once you have a local copy of the project on your device, open `trace.psyexp` in the PsychoPy Builder.

Welcome!


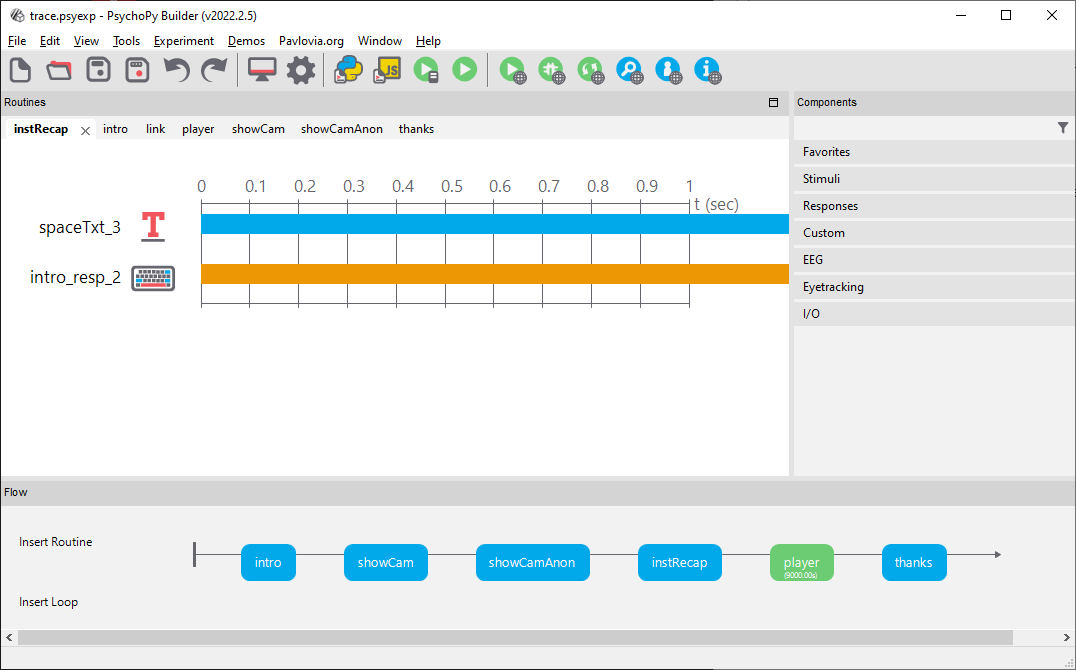


**Setting up**

You are looking at a basic experiment using Trace. The following sessions and code components are the minimum required for Trace to run and write data successfully.

**Experiment settings**

Trace requires `Save csv file (trial-by-trial)`. The option can be found under Edit Experiment Settings > Data. In the "Online" tab, face-api.js needs to be loaded.


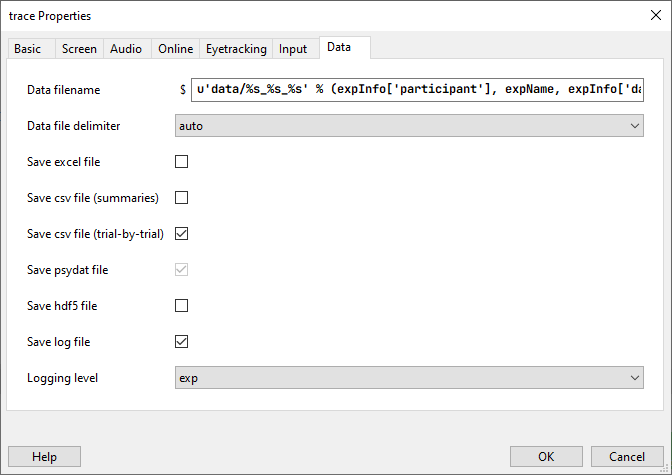


**Code Component 1: Initializing the faceDetector**

Located in the first Routine of the Experiment (“intro”), the Code Component (facedetectorSetup) initializes the face detector.

Select the `Begin Experiment` tab to view the initialization of the faceDetector. A full description of parameters and methods available for the faceDetector class is available at the PsychoJS API (https://psychopy.github.io/psychojs/FaceDetector.html).

First, we initialize an instance of the faceDetector class:
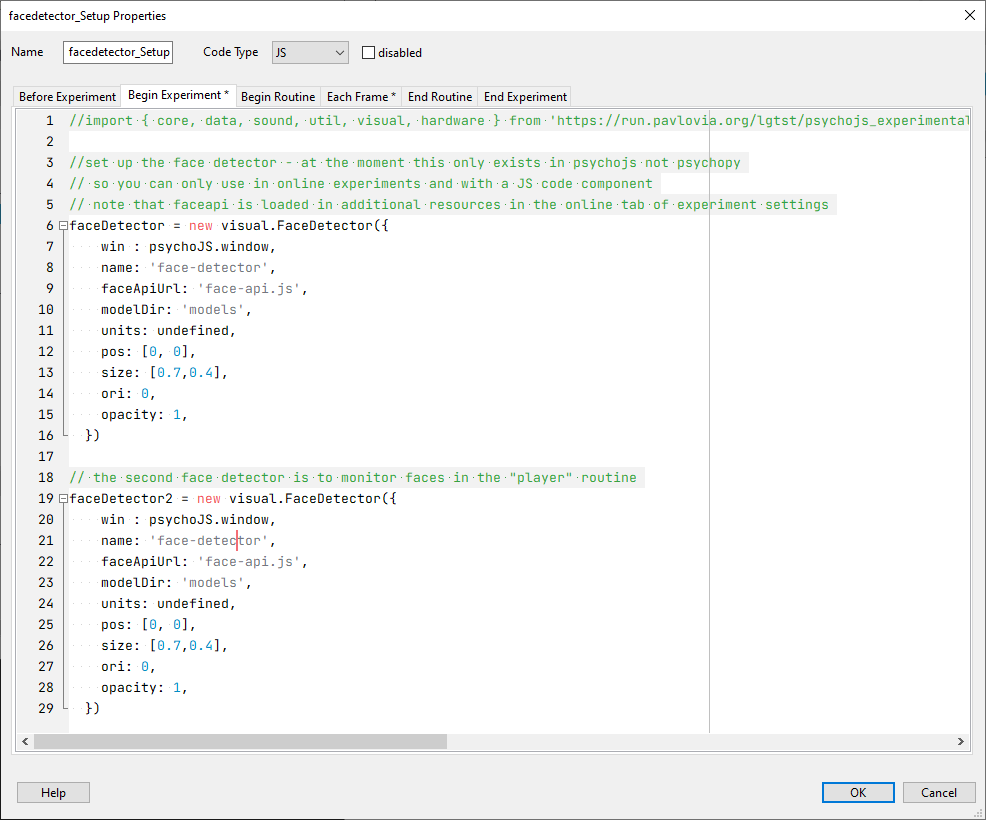


**win**: which window do you want to draw the faceDetector in (I.e. the face landmarks).

**name**: the name of the faceDetector object.

**faceApiUrl:** where can the face-api module be found (note we store the file “face-api.js” file in the same location as the .psyexp file and import it in experiment settings > Online > additional resources.

**modelDir:** subfolder location where model source code is stored.

**units**: What unit of measurement to use for drawing the faceDetector. If “units” are undefined, unit will be inherited from experiment settings. In this example, the experiment uses “height” units (indicating proportion of window height),

**pos**: The x and y coordinate to use as the central position for the faceDetector when drawn ([0,0] is the center of the screen, negative x/y values correspond to the left/down and positive to the right/up).

**size:** The size of the faceDetector when drawn. If you plan to show the face detector overlaying an webcam feed, it is a good idea to set the position and size of the face detecor to be the same as the dimensions used for the viewfinder (this is illustrated in the showCam routine of this Trace template). In this example, the experiment uses “height” units (indicating proportion of window height), the faceDetector is therefore set to be 70% of the window height in width and 40% of the window height in height, using height units means that Trace will scale the view finder and faceDetector appropriately to different screen sizes.

**ori**: orientation at which to draw the faceDetector in degrees.

**opacity**: the opacity of the faceDetector when drawn 0 = fully transparent 1 = fully visible

Under `Each Frame` we wait until the faceDetector has initialized and then end the intro routine. faceDetector.isReady() will return true when the faceDetector has been initialised.


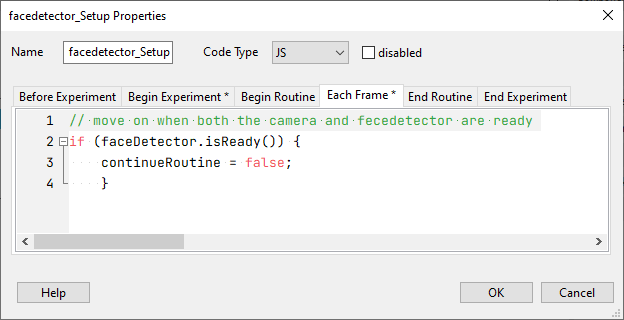


**Player Routine**

The Player routine presents a YouTube stream and records data from the faceDetector.
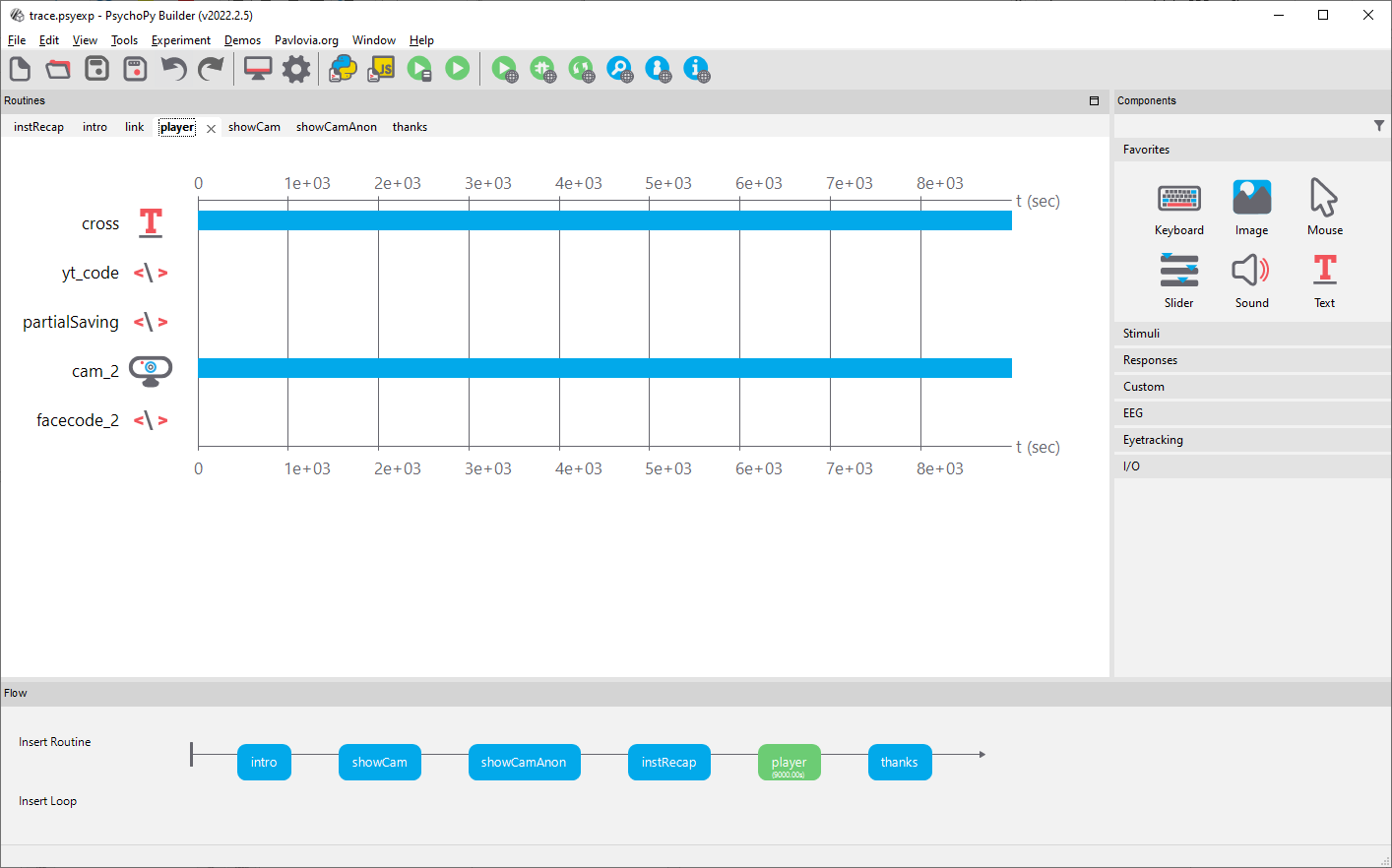


**Code Component 2: Start faceDetector Recording**

To record data from the faceDetector, we first need to provide an input to the detector. In this case we provide a webcam stream – which is the name of the webcam component in this routine “cam_2”.

faceDetector2.setInput(cam_2);

We then set up an asynchronous call to fetch and log data from the faceDetector, in addition to timestamps from the YouTube video. The start() function will indicate the detection period, in ms (e.g. 100 ms for 10Hz). We check if landmarks are being detected, and if so we fetch the youtube timestamp:

yt_t = movieStim.getPlaybackTime()

and save it to the data file under the column header “yt_time”:

psychoJS.experiment.addData('yt_time', yt_t);

We then also fetch the facial expression data:

happyDetection = detections[0]['expressions'].happy;

sadDetection = detections[0]['expressions'].sad;

neutralDetection = detections[0]['expressions'].neutral;

angryDetection = detections[0]['expressions'].angry;

disgustedDetection = detections[0]['expressions'].disgusted;

surprisedDetection = detections[0]['expressions'].surprised;

fearfulDetection = detections[0]['expressions'].fearful;

And store those to the data file using the psychoJS.experiment.addData() function.


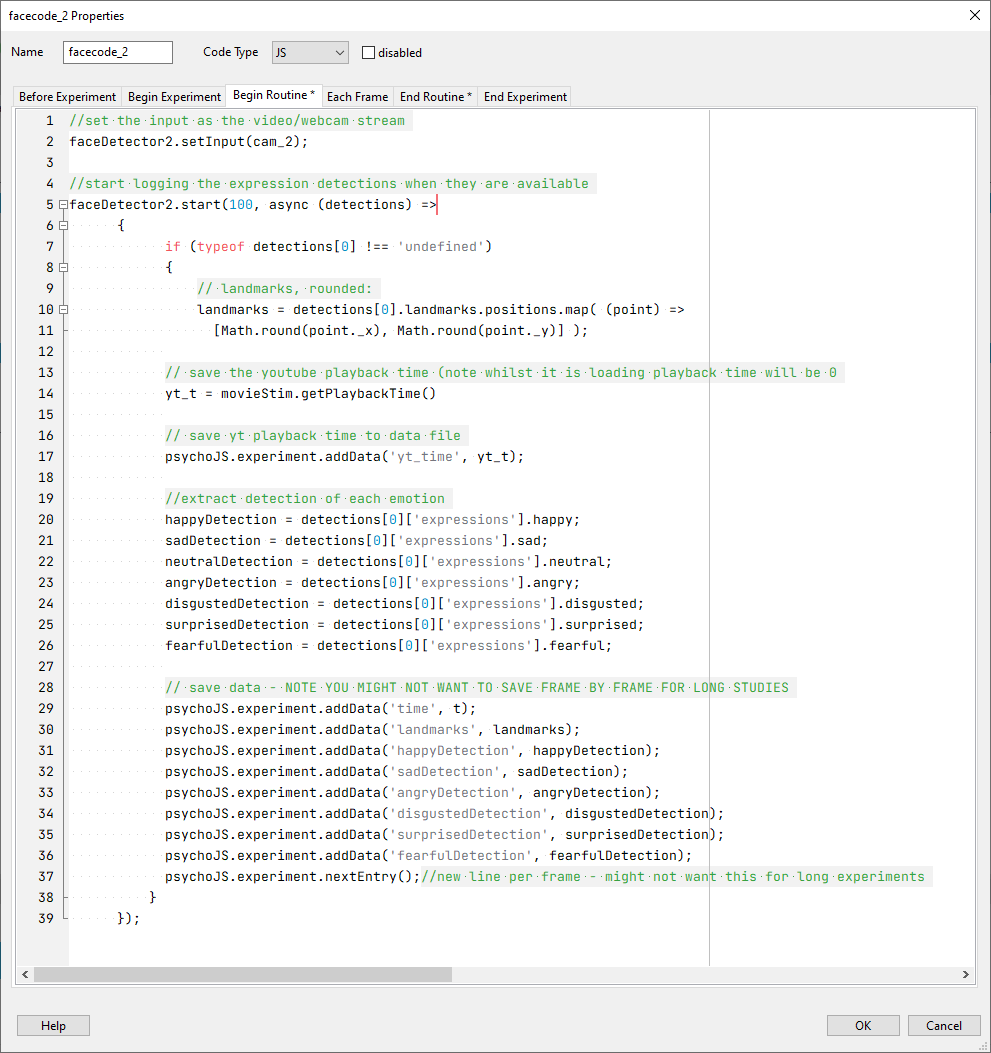


In the “End Routine” tab we then end the faceDetector.


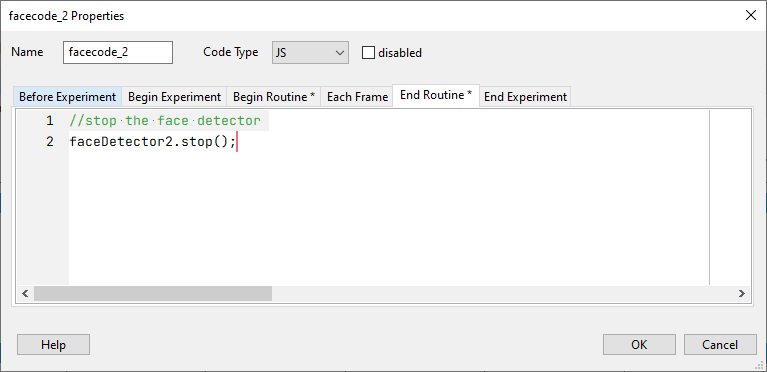


**Presenting stimuli**

Because Trace is developed within the PsychoPy/PsychoJS infrastructure, it can be used alongside any stimulus supported by this package, including images, text, movies and more (see <https://psychopy.org/api/index.html>). This documentation covers 3 ways of presenting movie-based content: A) Hosting internally within the experiment; B) Hosting on YouTube.

**A. Content hosted on Pavlovia**

**To use Trace with internally hosted content, simply add the content (e.g., Movie component) to the routine in which Trace is set up.**

**B. Content hosted on YouTube**

The Trace protocol was designed to work with YouTube API to deliver videos within the experiment. At present, YouTube streaming is supported via an extension of the movieStim class in PsychoJS, at time of writing this manuscript, this is a new feature, for which we interact with via code components (as opposed to using the movie component in PsychoPy Builder directly). Here we describe how to initialize a movieStim instance for streaming YouTube content. Initialization of the movieStim instance is demonstrated within the `Player Routine` of the template .psyexp file. The parameters are as follows:

**win**: which window do you want to draw the movie in.

**name**: the name of the movieStim object.

**youtubeURL:** the url to the YouTube content to stream.

**showYoutubeControls:** show the movie playback controls on the streamed video (true or false)

**disableYoutubePlayerKeyboardControls**: prevent the user from controlling movie playback via the keyboard (I.e. space for pause/play). True will prevent the user from using keyboard controls.

**units:** units to present the movie in (if undefined units are inherited from the experiment settings).


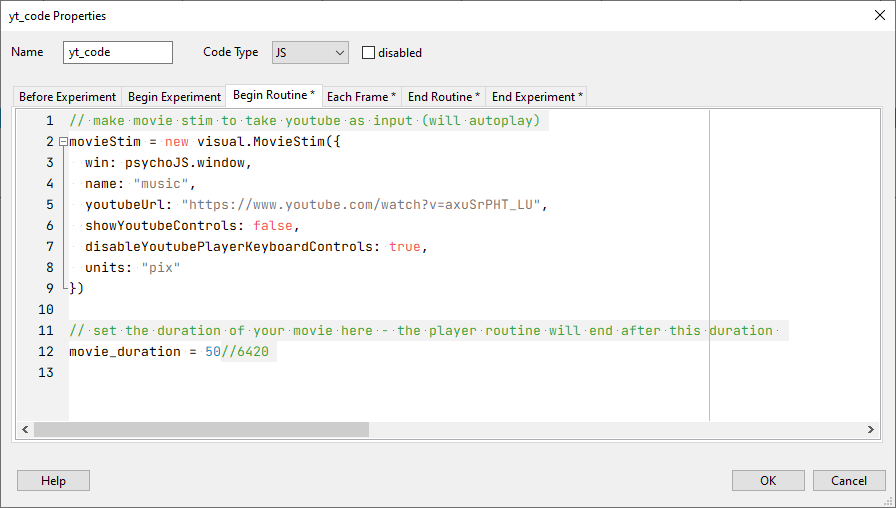


Additional methods available include,_ytPlayerIsReady which indicates when the movie is loaded and playing.

To end the routine when the movie has finished playing we check when time (t) exceeds a pre-defined duration (movie_duration) and then use the PsychoPy/JS method continueRoutine to end the current trial routine (I.e. end Player).


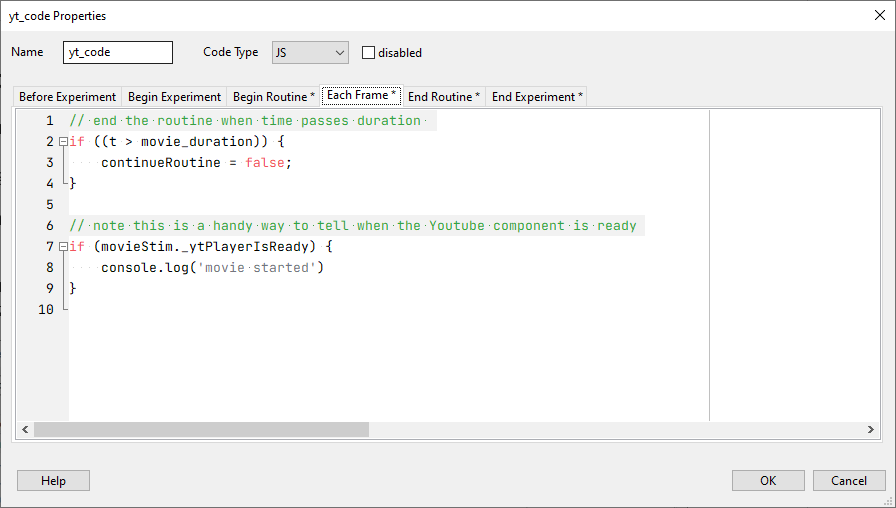
At the end of the routine, we can stop drawing the movie by setting the setAutoDraw method to false.


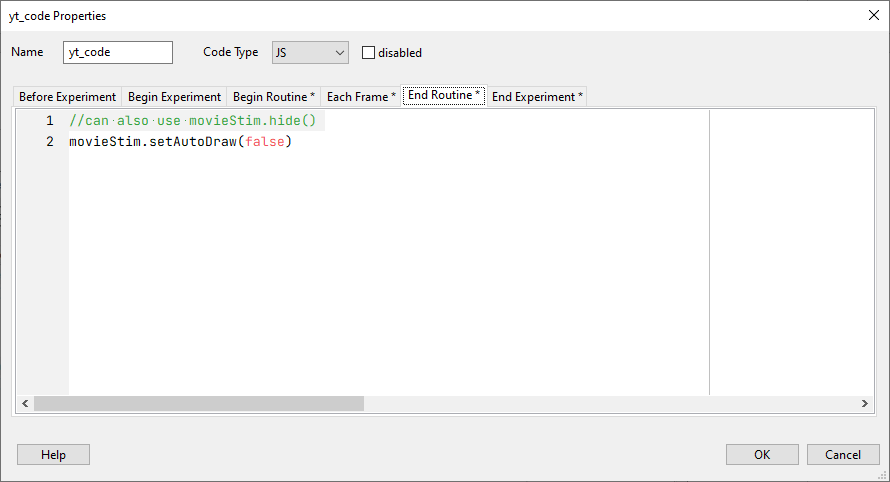


**B. Content hosted within experiment**

Please note that hosting within the experiment, i.e., on Pavlovia servers is discouraged, as it would result in a large experiment and potentially decrease performance.

**Deploying on Pavlovia**

Trace currently relies on a custom version of psychojs and an additional step is required to commit changes. However, this is rapidly being updated and will be implemented for the full release.

1. Generate a JS file using the JS icon in PsychoPy Builder. A PsychoPy Coder window with JS code should appear.
2. In the 5. line of code, change the content between the parentheses to: `./psychojs-yt.js`.
3. Commit to Pavlovia using the Sync icon.


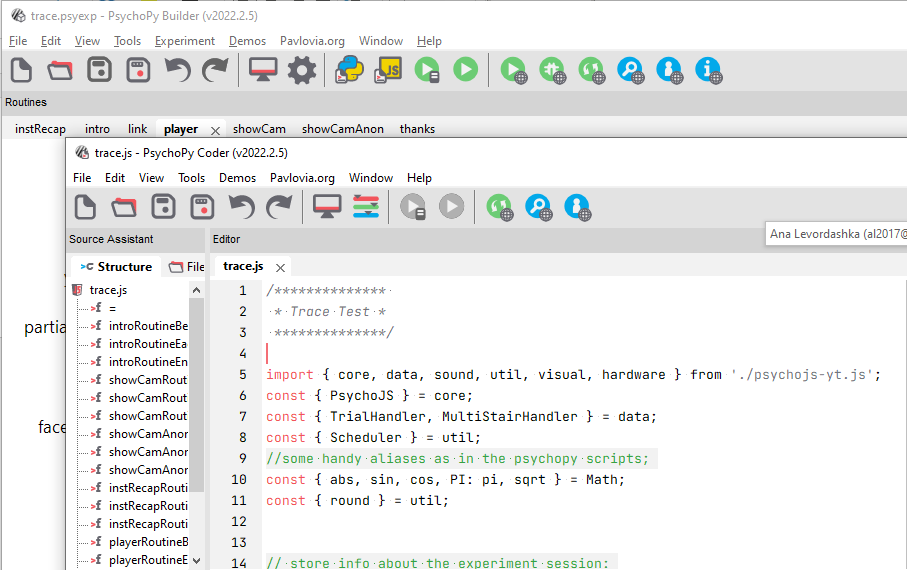


**3.**

**2.**

**1.**

**Data**

To ensure maximum retention, Trace outputs data in files, in csv format. The files contain participant ids and need to be merged. On rare occasions, duplication of rows may occur at merging. These can be resolved by removing duplicate rows by timestamp. Each row in the file should contain a unique timestamp.
